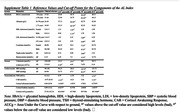# The associations between sleep disturbances and allostatic load, and the role of sex, beta amyloid, and burnout history in a memory clinic sample: the Co‐STAR study

**DOI:** 10.1002/alz.085618

**Published:** 2025-01-09

**Authors:** Dickson Olusegun Adedeji, Jasper Holleman, Ingemar Kåreholt, Alina Solomon, Miia Kivipelto, Shireen Sindi

**Affiliations:** ^1^ Geriatric Psychiatry Department Sanderud, Innlandet Hospital Health Trust, Ottestad, Hamar Norway; ^2^ Division of Clinical Geriatrics, Karolinska Institute, Stockholm Sweden; ^3^ Division of Clinical Geriatrics, Karolinska Institutet, Stockholm Sweden; ^4^ Aging Research Center, Karolinska Institutet, and Stockholm University, Stockholm Sweden; ^5^ Institute of gerontology, School of Health and Welfare, Aging Research network – Jönköping (ARN‐J), University of Jönköping, Jönköping Sweden; ^6^ Division of Clinical Geriatrics, Center for Alzheimer Research, Department of Neurobiology, Care Sciences and Society (NVS), Karolinska Institutet, Stockholm Sweden; ^7^ Theme Inflammation and Aging, Karolinska University Hospital, Stockholm Sweden; ^8^ Ageing Epidemiology Reseach Unit (AGE) Imperial College London, London UK; ^9^ Institute of Clinical Medicine, University of Eastern Finland, Kuopio Finland; ^10^ Department of Neurobiology, Care Sciences and Society, Division of Clinical Geriatrics, Center for Alzheimer Research, Karolinska Institutet, Stockholm/Solna Sweden; ^11^ The Ageing Epidemiology (AGE) Research Unit, School of Public Health, Imperial College London, London UK; ^12^ Ageing Epidemiology Reseach Unit (AGE), School of Public Health, Imperial College London, London UK; ^13^ Karolinska Institutet, Department of Neurobiology, Care Sciences and Society, Division of Clinical Geriatrics, Center for Alzheimer Research, Stockholm Sweden

## Abstract

**Background:**

Several studies have investigated the link between sleep disturbances and allostatic load (AL), but the results are varied, and less is known about the associations in clinical samples. The goal of this study is to assess the associations between sleep disturbances and AL among memory clinic participants, and to examine differences according to sex, beta‐amyloid status and history of burnout status.

**Method:**

The study was based on 146 memory clinic participants diagnosed with either Mild Cognitive Impairment (MCI) or Subjective Cognitive Impairment (SCI) in the Cortisol and Stress in Alzheimer’s Disease Study (Co‐STAR) (Sweden). Self‐reported sleep was measured using the Karolinska Sleep Questionnaire (KSQ). The AL index is a measure of chronic stress including blood‐based markers reflecting the neuroendocrine, immunological, metabolic, and cardiovascular systems, constructed using 16 biomarkers. Beta‐amyloid was measured from cerebrospinal fluid samples. History of burnout was self‐reported. Linear regressions were conducted to assess the associations between sleep disturbances and allostatic load, while adjusting for age, sex and sleep medications.

**Result:**

Lower total sleep score (b=‐0.228, p= 0.028) and sleep apnea index (b=‐0.187, p= 0.046) correlated with higher AL index. In stratified analyses, lower total sleep score was associated with higher AL index in women (b=‐0.282, p= 0.049), while higher insomnia levels (b=‐0.295, p= 0.038) were linked to lower AL levels in men. AL was only associated with daytime sleepiness in participants with normal beta‐amyloid levels, where a lower daytime sleepiness score (b=‐0.298, p= 0.038) was linked to higher AL levels. Among those without a history of burnout, higher AL index correlated with lower total sleep score (b=‐0.249, p= 0.045), insomnia score (b=‐0.223, p= 0.045), difficulty waking up score (b=‐0.225, p= 0.038), and non‐restorative sleep index (b=‐0.221, p= 0.043).

**Conclusion:**

This study shows varied associations between sleep disturbances and across different subgroups, highlighting the roles of age, sex, beta‐amyloid levels, and history of burnout in the associations. It may be of valuable importance to consider AL when evaluating sleep disturbances and implementing lifestyle interventions for the reduction of chronic stress and sleep disturbances to reduce the risk of cognitive decline.